# Functional connectivity in a monetary and social incentive delay task in medicated patients with schizophrenia

**DOI:** 10.3389/fpsyt.2023.1200860

**Published:** 2023-08-30

**Authors:** Bernd Hanewald, Denise Elfriede Liesa Lockhofen, Gebhard Sammer, Markus Stingl, Bernd Gallhofer, Christoph Mulert, Jona Ruben Iffland

**Affiliations:** Center for Psychiatry, Justus Liebig University Giessen, Giessen, Germany

**Keywords:** schizophrenia, monetary incentive delay (MID) task, social incentive delay (SID) task, functional connectivity, fMRI

## Abstract

**Introduction:**

Numerous studies indicate impaired reward-related learning in individuals with schizophrenia, with various factors such as illness duration, medication, disease severity, and level of analysis (behavioral or neurophysiological data) potentially confounding the results. Patients with schizophrenia who are treated with second-generation antipsychotics have been found to have a less affected reward system. However, this finding does not explain the neural dysfunctions observed in previous studies. This study aimed to address the open question of whether the less impaired reward-related behavior is associated with unimpaired task-related functional connectivity or altered task-related functional connectivity.

**Methods:**

The study included 23 participants diagnosed within the schizophrenia spectrum and 23 control participants matched in terms of age, sex, and education. Participants underwent an MRI while performing a monetary incentive delay task and a social incentive delay task. The collected data were analyzed in terms of behavior and functional connectivity.

**Results:**

Both groups exhibited a main effect of reward type on behavioral performance, indicating faster reaction times in the social incentive delay task, but no main effect of reward level. Altered functional connectivity was observed in predictable brain regions within the patient group, depending on the chosen paradigm, but not when compared to healthy individuals.

**Discussion:**

In addition to expected slower response times, patients with schizophrenia demonstrated similar response patterns to control participants at the behavioral level. The similarities in behavioral data may underlie different connectivity patterns. Our findings suggest that perturbations in reward processing do not necessarily imply disturbances in underlying connectivities. Consequently, we were able to demonstrate that patients with schizophrenia are indeed capable of exhibiting goal-directed, reward-responsive behavior, although there are differences depending on the type of reward.

## Introduction

The pathomechanism of schizophrenia remains incompletely understood; however, a significant finding is the increase in dopamine in the limbic system (1). At the same time, dopamine plays a pivotal role as a neurotransmitter in the reward system (2). The ability to anticipate potential rewards is essential for developing motivation toward reward-driven behavior. Consequently, the learning process of associating environmental stimuli with rewarding events is a critical determinant of goal-directed behavior and motivation (3).

Given that motivational deficits significantly impact the quality of life, and numerous studies indicate impairments in reward-related learning among individuals with schizophrenia (4, 5), along with the limited effectiveness of common drug treatments, reward processing in schizophrenia represents an area of particular clinical interest. Various potential explanations exist for these motivational impairments, including deficits and irregularities in dopamine transmission (1, 6–8). It should be emphasized that regarding motivational deficits in schizophrenia, patients generally exhibit relatively intact consummatory pleasure (“liking”) but limited motivation to attain a reward (“wanting”) (9–11). This may be associated with deficits in orbitofrontal cortex-driven value representation and difficulties in “effort-cost” computation (12). In the latter case, an overexpression of postsynaptic D2 receptors (rather than reduced striatal dopamine release), along with cingulate dysfunction, may contribute to the aberrant computation of effort value in patients with schizophrenia (5, 13).

In individuals suffering from schizophrenia, the specific “stimulus-linked release of dopamine that mediates (…) the expression of appropriate motivational saliences (…) is dysregulated to a stimulus-independent release of dopamine” (14). Consequently, distinguishing between important and unimportant environmental stimuli appears to be more challenging for patients with schizophrenia, resulting in “aberrant salience” toward irrelevant stimuli and reduced salience attribution to cues predicting rewards (14).

Studies investigating reward processing in schizophrenia present a diverse landscape influenced by various factors, including illness duration (15), medication (16–20), disease severity and stage (15), and the level of analysis, such as behavioral (21) or neurophysiological data (12).

At the neural level, disrupted reward-related learning in schizophrenia is associated with abnormalities in ventral striatal activation (22, 23) and anomalies in the midbrain, insula, and amygdala (16, 24–27). For instance, individuals with schizophrenia exhibit reduced activation in the ventral striatum (VS), including the nucleus accumbens (NAcc), in response to prediction errors (23). Moreover, patients display excessively stronger VS activations when presented with neutral stimuli than healthy controls (22, 28). These findings suggest that alterations in the mesolimbic dopamine system underlie deficits in reward-based learning.

In addition to these findings, Gradin et al. (25) propose that schizophrenia is more related to abnormal interactions between brain systems, rather than focal brain abnormalities. A review on disconnectivity in schizophrenia reveals two prominent trends across different stages of the disorder: reduced connectivity and frequent involvement of frontal regions (29). Altered connectivity has been observed within a brain system encompassing the insula and the anterior cingulate cortex (ACC) (30), and the interaction between the insula–ACC system and the reward system may account for reported irregularities in reward processing studies (25). Furthermore, another dopamine-associated circuit appears to be dysregulated in schizophrenia, leading to cognitive impairments, deficits in emotional processing, and motor dysfunctions (31). Evidence suggests that the cerebellum plays a role in higher cortical functions (32, 33). Consequently, Parker et al. (34) propose the existence of a cingulo-cerebellar circuit (CCC), involving the thalamus and the ventral tegmental area (VTA), which connects the cerebellum and the ACC through afferent and efferent pathways. Tracing studies have demonstrated the CCC and indicate functional and structural abnormalities underlying schizophrenia (35–37). Within the CCC, the cerebellum's impact on decision-making, pattern perception, and error detection is mediated by inhibitory Purkinje cells and excitatory granule cells. These aforementioned alterations and aberrant connections could contribute to cognitive deficits and inefficient reward processing in individuals with schizophrenia (38, 39). Furthermore, the ACC is implicated in normal cognition and executive functions, including emotional processing, working memory, attention, response inhibition, performance monitoring, and timing (34, 40). Additionally, the cerebellum has been found to be activated during various cognitive tasks, even when motor activation is controlled for. These include, but are not limited to, face recognition, emotion attribution, and different types of memory (32, 41–43). Aberrant connections between perceptions and their meanings can arise from erroneous connections from the cerebellum to the cerebral cortex, resulting in errors in perceptual processing and misinterpretations (34).

In recent years, there has been a significant increase in the number of studies investigating human reward processing, encompassing both behavioral and neuroimaging approaches, using various types of incentives such as monetary (44) and social rewards (45–48). In these studies, reward is defined as the positive and contingent outcome of successful behaviors, with the potential to increase the likelihood of certain behaviors in the long run (49).

A considerable body of research has examined the human brain's sensitivity to anticipate rewards based on promising cues, such as food (50), monetary rewards (44, 51), or social stimuli (47). Spreckelmeyer et al. (47) introduced the social incentive delay paradigm (SID), which is a modified version of the classic monetary incentive delay paradigm (MID) initially developed by Knutson et al. (44, 51). In the SID, successful behavior is rewarded by appealing faces, instead of monetary units.

In our previous study, we investigated a reward paradigm, including both monetary (MID) and social stimuli (SID), in a sample of 54 patients with schizophrenia and 54 comparable control subjects (52). We found faster reaction times in the SID task than in the MID task, and there were no significant differences in the behavioral responses of patients between the two tasks. The majority of patients were receiving treatment with second-generation antipsychotics (SGAs) and were able to differentiate between reward levels in both paradigms. When comparing patients with control subjects, no discernible impairments were observed at the behavioral level, except for slower reaction times and lower hit rates. The patient group did not exhibit significant deficits in “wanting” on the behavioral level, which refers to the desire and longing for a reward.

These findings suggest that patients with schizophrenia are capable of exhibiting reward-oriented behavior and anticipating potential rewards. This aligns with the findings of Schlagenhauf et al. (18), who reported a less impaired reward system in patients with schizophrenia treated with SGAs. However, these findings do not fully explain the neural disturbances observed in previous studies, such as abnormal cortical–striatal interactions, bilateral hypoactivation in the VS during reward anticipation, or deficits in dorsolateral prefrontal cortex function (12, 15, 21). Therefore, it is crucial to further investigate potential neurofunctional impairments in social and monetary reward processing.

As discussed above, studies have indicated altered functional connectivity in patients with schizophrenia compared with healthy controls, including reward processing. However, current research on behavioral data related to reward processing shows minimal differences between patients treated with SGAs and healthy control participants. This study aimed to address the open question of the relationship between less impaired reward-related behavior and the underlying task-related functional connectivity in schizophrenia.

## Methods

### Participants

The study sample consisted of 23 participants diagnosed within the schizophrenia spectrum (SZ) and 23 control participants (CS) who were paired based on age, sex, and education. The SZ group was recruited from two psychiatric hospitals in Giessen, Hesse, Germany, and comprised of post-acute inpatients. Diagnoses were established using the Structured Clinical Interview for DSM-IV Axis I disorders (53) and available medical records. Symptom severity was assessed using the *Positive and Negative Syndrome Scale* [PANSS; (54)]. Trained psychiatrists and clinical psychologists performed the diagnostic assessments. Patients who met any of the following exclusion criteria were not included: intellectual disabilities (IQ < 70), severe neurological disorders, acute self-endangerment or endangerment of others, organic psychotic disorders, pharmaceutical or drug-induced psychotic disorders, and insufficient comprehension of the German language.

The CS group was drawn from a community sample and recruited through mailing lists, social media, newspaper ads, and posted notices in shops. Individuals were excluded if they had ever been treated for schizophrenia, had received psychiatric or psychotherapeutic treatment within the last 6 months, or had a first-degree relative with schizophrenia.

Demographic, psychopathological, and medication-related characteristics of both study groups are presented in Table 1. Patients with schizophrenia exhibited minimal levels of positive symptoms and mild levels of negative symptoms. The majority of patients were treated with second-generation antipsychotics (SGAs), with a mean chlorpromazine-equivalent dosage of 631.0 mg per day (55).

**Table 1:** Demographic, psychopathological, and medicinal characteristics for SZ and CS.

	**SZ (*N* = 23)**	**CS (*N* = 23)**
Sex (N, male/female)	12/11	12/11
Age (in years)	35.3 (10.5)	35.1 (11.2)
Duration of illness (in years)	12.1 (9.8)	—
**Psychopathology**		
**PANSS original scales**		
PANSS total	61.2 (14.1)	—
PANSS positive	13.4 (5.6)	—
PANSS negative	18.0 (5.2)	—
PANSS general	29.9 (6.5)	—
**Medication**		
CPZ	631.0 mg (336.6 mg)	—
FGA + SGA	*n* = 3	—
FGA + SGA + SGA	*n* = 2	—
SGA	*n* = 13	—
SGA + SGA	*n* = 5	—

N, Number of participants.

PANSS, Positive and Negative Syndrome Scale; CPZ, chlorpromazine equivalent; FGA, first-generation antipsychotic; SGA, second-generation antipsychotics; FGA + SGA, combined treatment with FGA and SGA; FGA + SGA + SGA, combined treatment with one FGA and two SGA; SGA + SGA, combined treatment with two SGA.

The study received approval from the Ethics Committee of the Medical Faculty of the University of Giessen in accordance with the principles outlined in the Declaration of Helsinki. All participants provided written informed consent prior to participating in the study. The sample size with G^*^Power recommended a total sample size of 30 participants (repeated measurements within–between interaction (ANOVA): effect size partial eta-squared = 0.3, α = 0.05, 1-ß power = 0.9, repeated measurements = 2, number of groups 2). The option for effects size specification was set to “as in SPSS” as recommended by Aberson et al. (56).

### Stimuli and task

The experiment comprised two distinct tasks, namely, the monetary incentive delay task (MID) (44) and the social incentive delay task [SID, (47)]. Each task consisted of 88 trials, divided into two blocks, resulting in a total of four blocks with 44 trials each.

**Figure 1:**
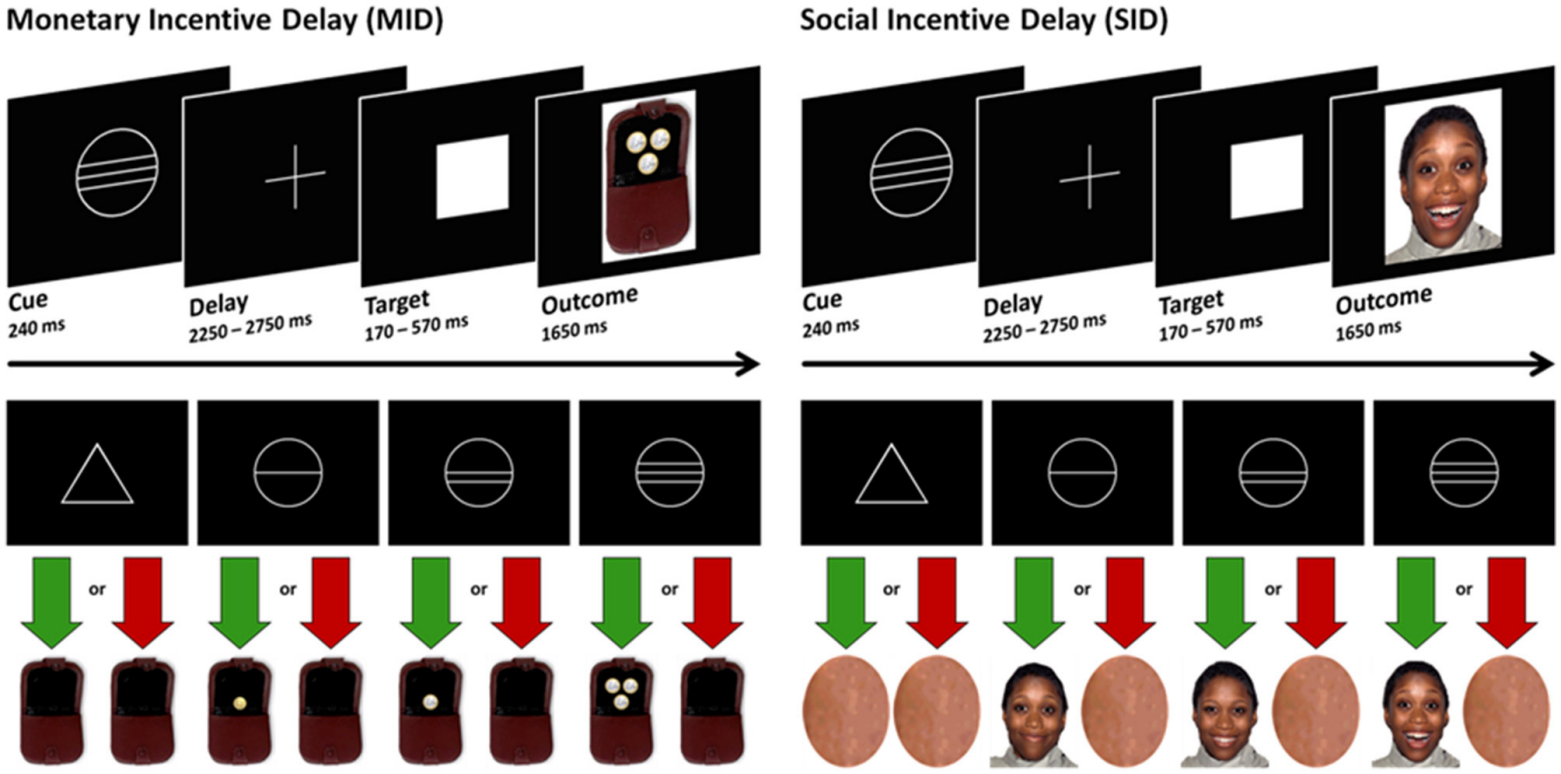
Experimental paradigm.

Participants completed these four blocks (two MID, two SID) in a single run, with the reward condition alternating between blocks. The order of the tasks was counterbalanced across participants. At the beginning of each task, participants received instructions regarding which task (MID or SID) they would be performing. Each trial began with a cue lasting for 240 milliseconds, followed by the presentation of a crosshair (ranging from 2,250 to 2,750 ms), the target symbol (individually adjusted presentation time, ranging from 170 to 570 ms), and the feedback screen (1,650 ms, see Figure 1). The feedback, representing the reward outcome, depended on whether participants were able to press a button within an individually adjusted time window after the appearance of the target symbol (white square) on the screen. Prior to the experiment, the time window for the reaction (target duration) was adjusted based on a single reaction time task to accommodate each participant's reaction speed.

In both the MID and SID paradigms, there were three levels of potential reward, as well as a neutral outcome, signaled by cues that participants learned during a training session before the experiment. Circles indicated potential rewards (66 trials per task), while triangles indicated no expected reward (22 trials). The number of horizontal lines within each circle represented one of three levels of potential reward in the MID task (€0.20, €1.00, and €3.00) and in the SID task [three happy facial expressions with increasing intensity; for further details, see Spreckelmeyer et al. (47)]. Provided that a timely response occurred within the designated time window, feedback was given in the form of pictures showing a happy face or a wallet displaying the virtually earned amount of money. In case of a slow response or no response, an empty wallet or a graphically distorted face lacking any facial features was displayed [for additional details, see Spreckelmeyer et al. (47)]. Trial categories were presented in a pseudo-random order within the MID and SID sessions. The inter-trial intervals were jittered, ranging between 2,500 and 5,000 ms.

### Analyses

#### Behavioral data

Statistical analyses were performed using SPSS version 22 (57). Mean reaction times were calculated averaging the medians of the responses of each single subject regarding different reward levels and conditions. Mean hit rates resulted from the number of correct trials (responses in time) of each subject regarding different reward levels and conditions. Reaction times and hit rates were analyzed using a 2 × 2 × 4 analysis of variance (ANOVA). The between-subject factor was “*group”* (SZ, CS) and the within-subject factors were “*reward type*” (monetary, social) and “*reward level*” (no reward, low, medium, and high reward). *F*-values and Greenhouse–Geisser corrected *p*-values are reported, and squared eta-correlation coefficients (η^2^) refer to effect sizes. In the case of statistically significant interactions (*p* < 0.05), *post-hoc* analyses between reward levels within each group were performed.

### MRI-data

#### Data acquisition

Both structural and functional data were collected during the MRI examination. The participants were prepared for the MRI examination outside of the MRI scanner; in addition to observing safety precautions, all participants completed a trial run of the fMRI paradigm to ensure correct performance. After a 5-min anatomical measurement, the functional measurement was performed with the reward paradigm (approximately 30 min). The T2^*^-weighted EPI sequences were applied in a 3 T MRI (Siemens Verio; TR = 2.1 s; TE = 30 ms; flip angle = 90°; slice thickness = 4 mm; field of view (FoV) = 192 × 192 mm; matrix = 64 × 64 mm; voxel size = 3 × 3 × 5 mm).

### Analysis of “functional connectivity”

The task-based functional connectivity was analyzed using the CONN functional connectivity toolbox (58). A CompCor noise reduction method was implemented, which removes principal components attributed to white matter and cerebrospinal fluid (59) without the biases associated with global signal regression (60). Additionally, the six realignment parameters and their first-order derivatives, as well as the main effects of the task, were introduced as confounds (61). A temporal filter of [0.008 Inf] was applied to the resulting residual time series. Following the temporal preprocessing, functional connectivity measures (bivariate correlation) were computed between each pair of ROIs (ROI-to-ROI analysis). In total, 106 cortical and subcortical ROIs were derived from the Harvard-Oxford maximum-likelihood cortical atlas (62–65) and were included in the analysis. Subsequently, a Fisher transformation was applied to allow for parametric testing. The resulting correlation maps were carried forward to the group-level analysis, where the network-based statistics (NBS) method (66) was used to identify functional subnetworks that were differentially connected between the reward types (SID_reward_ > MID_reward_) and both groups (SZ > CS, CS > SZ). NBS uses a cluster-based approach to deal with the multiple comparisons problem arising from mass-univariate testing at every network connection. A primary threshold of p = 0.05 (FDR-corrected) was applied to the test statistic (two-sample *t*-test) to define a set of suprathreshold links. Any connected components and their number of links (size) were then identified. Using permutation testing, the FWE-corrected *p*-value was computed for each component based on its size. For a more detailed description of this procedure, refer Zalesky et al. (66). The comparison of the reward types was analyzed for both groups separately, and then, the data of both groups were compared. An α of < 0.05 was accepted as significant.

## Results

### Behavioral data

#### Reaction times

An ANOVA was performed to examine the impact of *reward level* and *reward type* on reaction times in both groups. The results showed a significant main effect of *reward type* [*F*_(1.0, 4.0)_ = 6.1, *p* < 0.05, η^2^= 0.12], indicating that participants had faster reaction times in the SID task than the MID task. Regarding the between-subject factor of *group*, a significant effect was found [*F*_(1.0, 44.0)_ = 6.3, *p* < 0.05, η^2^= 0.13], indicating that control participants (CS) exhibited significantly faster reaction times than participants with schizophrenia (SZ). However, there was no significant main effect of *reward level*, and no significant interactions were observed between *reward type* x *reward level, reward type* x *group*, and *reward level* x *group*.

**Figure 2:**
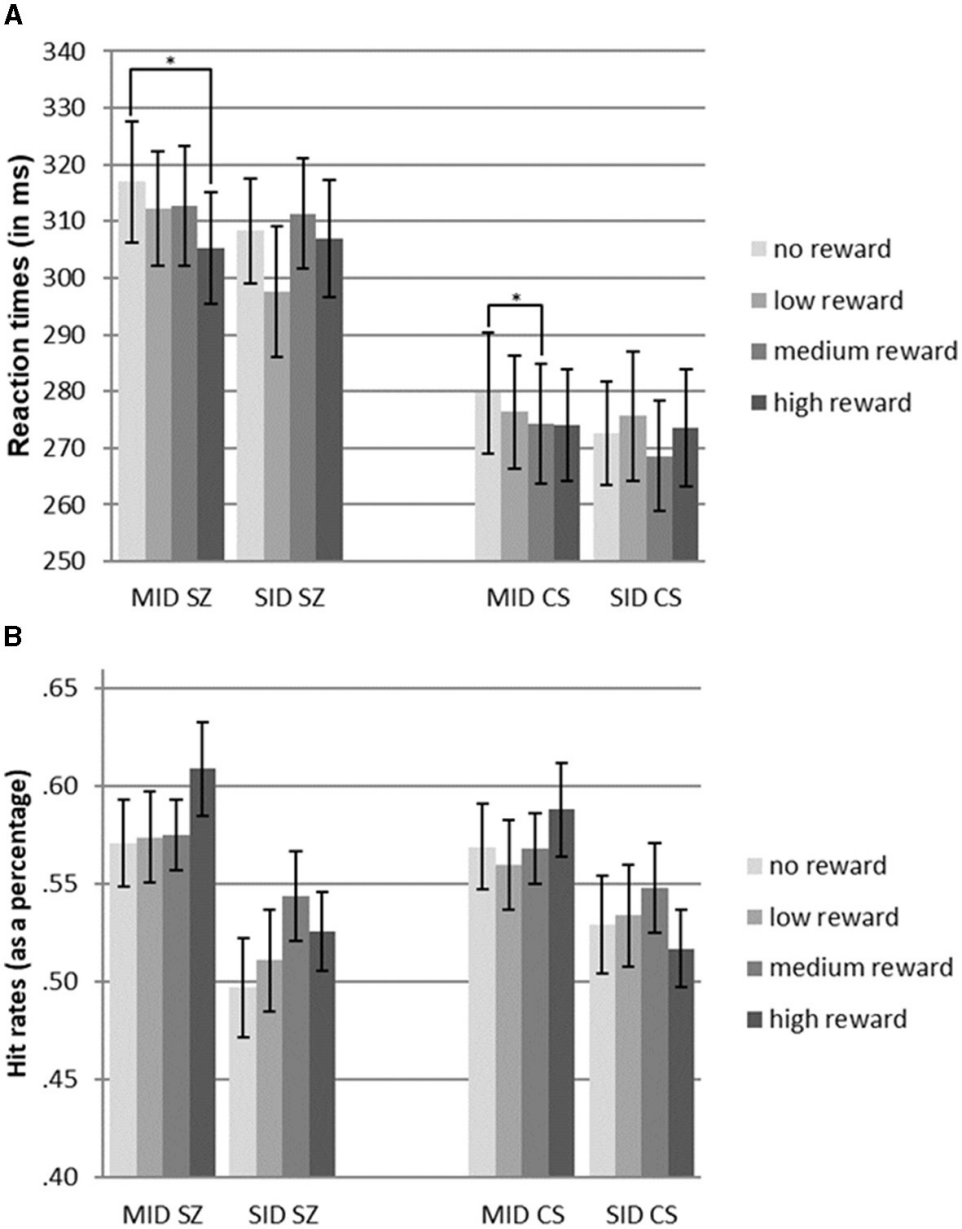
**(A)** Reaction times of patients diagnosed with schizophrenia (SZ) and community sample (CS) for monetary incentive delay tasks (MID) and social incentive delay tasks (SID). **(B)** Hit rates of patients diagnosed with schizophrenia (SZ) and the community sample (CS) for monetary incentive delay tasks (MID) and social incentive delay tasks (SID). Error bars indicate standard error (S.E.). Significant comparisons of means within the group and reward type are indicated by asterisks (pairwise *t*-test: *p < 0.05).

Further analysis using pairwise *t*-tests within the MID task showed significant differences in reaction times between high-reward and no-reward conditions for SZ [*t*_(22)_ = 2.58, *p* < 0.05], indicating faster reaction times for high-reward trials. Similarly, within the MID task, CS showed significant differences in reaction times between medium-reward and no-reward conditions [*t*_(22)_ = 2.33, *p* < 0.05], with faster reaction times for medium-reward trials. However, no significant differences between reward levels were found in the SID task (see Figure 2A, Table 2).

**Table 2:** Mean reaction times, mean hit rates, and averages for both reward types.

	**SZ (*****N*** = **23)**					**CS (*****N*** = **23)**				
	**Reward level**					**Reward level**				
	**0**	**1**	**2**	**3**	∅	**0**	**1**	**2**	**3**	∅
**Reaction times**										
MID	317.0 (66.3)	312.2 (62.1)	312.7 (67.0)	305.3 (60.7)	311.8	279.7 (28.9)	276.4 (27.8)	274.3 (23.4)	274.0 (28.8)	276.1
SID	308.3 (59.4)	297.5 (73.5)	311.3 (62.5)	307.0 (66.0)	306.0	272.6 (19.5)	275.6 (26.8)	268.6 (20.5)	273.6 (22.4)	272.6
**Hit rates**										
MID	57.1 (11.7)	57.4 (12.5)	57.5 (9.8)	60.9 (14.0)	58.2	56.9 (8.9)	56.0 (9.7)	56.8 (6.9)	58.8 (8.5)	57.1
SID	49.7 (13.5)	51.1 (13.9)	54.4 (13.6)	52.6 (10.0)	52.0	52.9 (10.4)	53.4 (11.0)	54.8 (7.9)	51.7 (8.7)	53.2

Mean reaction times (in ms; standard deviation) and hit rates (in percentages; standard deviation).

#### Hit rates

An ANOVA was performed to assess the impact of reward level and reward type on hit rates in both groups. The results indicated a significant main effect of *reward type* [*F*_(1.0, 44.0)_ = 17.9, *p* < 0.001, η^2^ = 0.29], indicating higher hit rates in the MID task than in the SID task. However, there were no significant main effects of *reward level* or *group*, suggesting comparable hit rates between participants with schizophrenia (SZ) and control participants (CS). Additionally, no significant interactions were found between *reward type* x *reward level, reward type x group*, and *reward level x group* (see Figure 2B, Table 2).

### Functional connectivity

#### Patients

**Figure 3:**
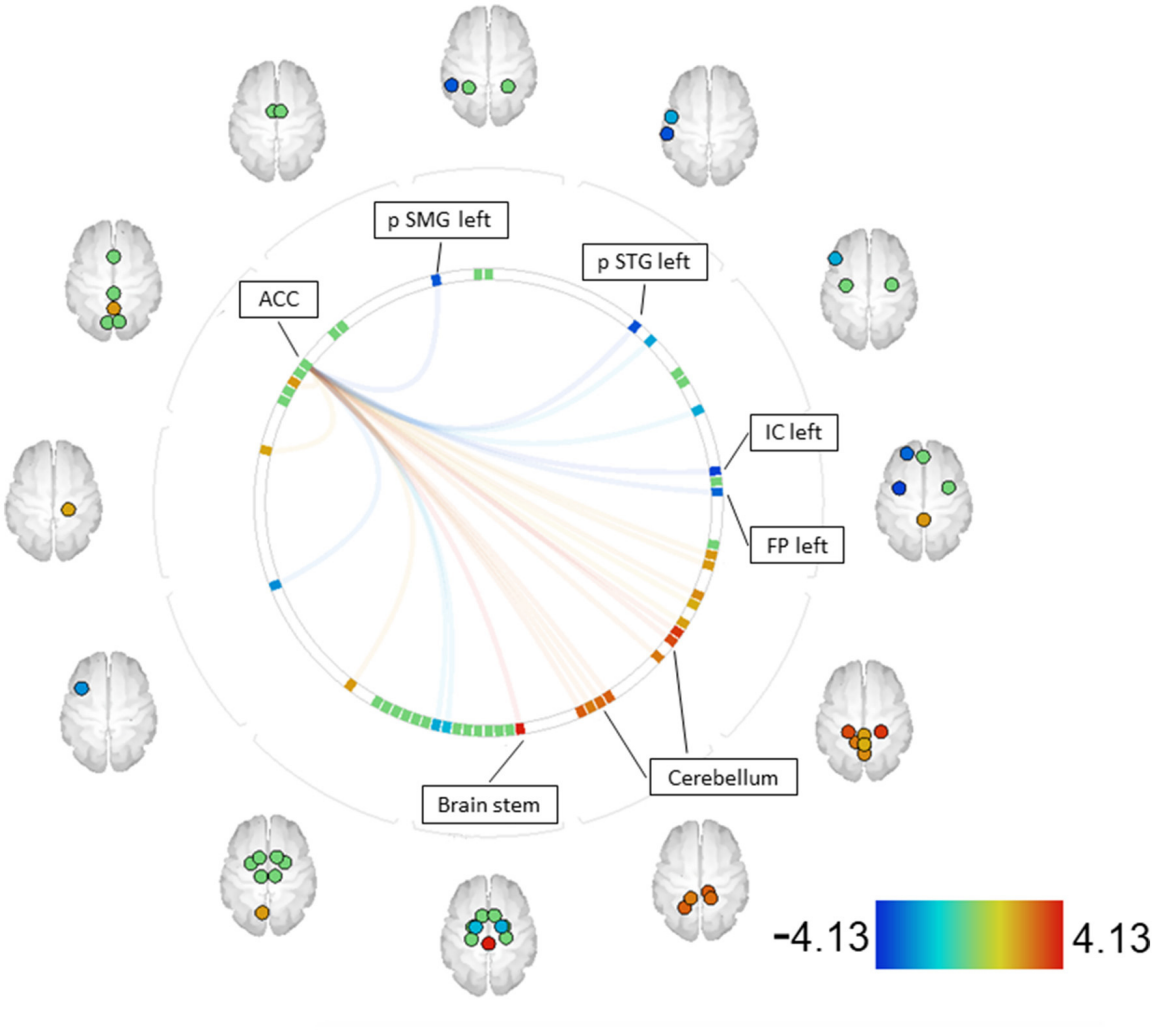
SZ MID > SID [functional network connectivity (FNC); seed analysis, ROI-to-ROI connections; connectome ring display]. Only ROIs with a positive or negative significant connection to one of the seeds (p-FDR < 0.05) are labeled. Seeds with significant connections: ACC [ACC—anterior cingulate cortex, FP—frontal pole, IC—insular cortex, pSMG—posterior supramarginal gyrus, pSTG–posterior supratemporal gyrus].

For the contrast MIDreward > SIDreward, increased couplings were observed between the ACC and the brain stem, as well as the cerebellum (right and left parts). Decreased couplings were found between the ACC and the posterior supramarginal gyrus (SMG) (left), the posterior superior temporal gyrus (STG) (left), the insular cortex (IC) (left), and the frontal pole (FP) (left) (see Figure 3, Supplementary material A).

#### Community sample

Regarding the contrast MIDreward > SIDreward, no significant differences in couplings were observed.

#### Patients vs. community sample—MID

**Figure 4:**
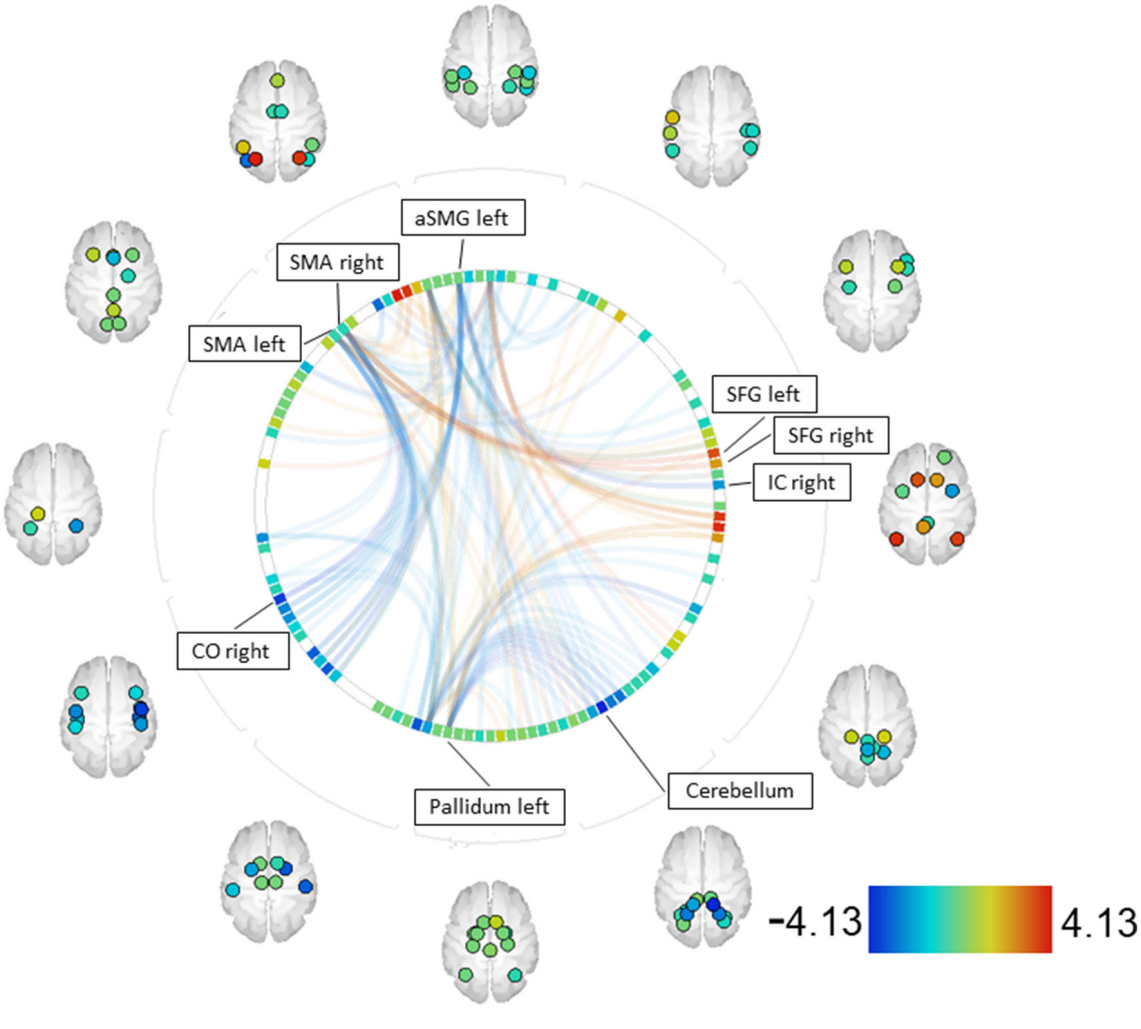
SZ > CS—MID [functional network connectivity (FNC); seed analysis, ROI-to-ROI connections; connectome ring display]. Only ROIs with a positive or negative significant connection to one of the seeds (p-FDR < 0.05) are labeled. Seeds with significant connections: SMA right, aSMG left, pallidum left, SMA left [CO—central opercular cortex, IC—insular cortex, SFG—superior frontal gyrus, SMA—supplementary motor areal, aSMG—anterior supramarginal gyrus].

When comparing patients with SZ to the community sample (CS) using the MID paradigm, the contrast SZ > CS revealed increased couplings between the right and left supplementary motor area (SMA) and the right and left superior frontal gyrus (SFG). Decreased couplings were observed between the right and left SMA and the right central opercular cortex (CO), as well as between the left pallidum and the right and left cerebellum (45). Furthermore, decreased couplings were found between the left anterior supramarginal gyrus (aSMG) and the IC (see Figure 4, Supplementary material B).

#### Patients vs. community sample—SID

When comparing patients with SZ to the community sample using the SID paradigm, no significant differences in couplings were observed.

## Discussion

### Behavioral data

This study aimed to investigate the response behavior and functional connectivity of individuals with schizophrenia (SZ) compared with matched control subjects (CS) during the anticipation of monetary and social rewards. It is well documented that SZ individuals exhibit slower reaction times and lower hit rates (67, 68). As expected, on the behavioral level, SZ participants demonstrated slower reaction times than CS participants in both the MID and SID tasks, while no significant differences were found in hit rates between the two groups.

For both groups, a main effect of reward type was observed, indicating faster reaction times in the SID task than in the MID task, but no significant main effect of reward level was found. These findings differ from those of Spreckelmeyer et al. (47) who reported faster reaction times in the MID task than in the SID task, as well as a decrease in reaction times with increasing reward levels in both tasks. Within the patient group, *t*-tests revealed significantly faster responses in the MID task when comparing high-reward to no-reward conditions. However, patients responded equally quickly during the SID task across all reward levels. This suggests that there was stronger discrimination of cue stimuli in the more abstract monetary reward condition than in the social reward condition. Interestingly, patients with schizophrenia were capable of graded responses depending on the cue stimulus, but these differences were evident depending on the specific paradigm used. In the SID task, various faces with different genders and emotions were presented, which may have contributed to faster reactions than in the MID task, where the monetary stimuli were less diverse. The social reward stimuli appeared to be more immediate and salient to participants than the monetary incentives (see Figure 1).

### Functional connectivity

#### Patients

Given the expected differences in discriminatory abilities between patients and control participants, it was reasonable and necessary to compare the underlying functional connectivity. In addition to the differences in reward anticipation between SZ and CS discusse above, to the best of our knowledge, no previous research has compared reward anticipation using different paradigms within the SZ population. Therefore, it was initially unexpected to find differences in functional connectivity between the two paradigms used within the patient group, which aligns with the behavioral differences discussed above. Increased connections were observed between the ACC and regions of the cerebellum in contrast to MIDreward > SIDreward. These findings suggest that dysfunctions may occur in the CCC, depending on the specific task being processed. In conjunction with behavioral data showing decreasing reaction times across MID reward levels, increased functional connectivity in the CCC might explain the patients' ability to discriminate stimuli and exhibit reward-oriented behaviors during the MID task. The CCC appears to respond differently depending on the underlying paradigm, which also corresponds to the behavioral differences observed, resulting in better discrimination of cue stimuli in the MID task.

Furthermore, decreased couplings were found between the ACC and the left posterior SMG, left posterior STG, left IC, and left FP within the MID task. The decreased couplings between the ACC and the left posterior SMG may suggest that the involvement of SMG in encoding postures and gestures of other people and being empathetic is not demanded during the MID task (69–71).

The decreased couplings between the ACC and the left posterior STG in the MID task compared with the SID task seem plausible, as the posterior STG is an important structure in the pathway involving the amygdala and prefrontal cortex, which are all involved in social cognition processes. The STG is involved in the recognition of emotions in facial stimuli (72, 73) and is associated with processing information about the various changeable characteristics of a face (72).

The decreased couplings between the ACC and the left IC and FP, which are part of the orbitofrontal cortex (OFC), suggest a role in motor adaptation after reward and could be jointly responsible for the overall faster reaction times observed in the SID task than in the MID task.

In the MID task, areas associated with stimulus discrimination, such as the CCC, show greater activation, whereas, in the SID task, increased activations were found in reward-associated areas, especially those involved in social cognitive processes, such as the left posterior STG.

#### Community sample

Despite observing comparable reaction patterns and a main effect of reward type on the behavioral level in both groups, the functional connectivity analysis did not reveal differences in the intensity of couplings between the SID and MID tasks for the CS. Therefore, we can assume that stimulus discrimination and reward processing require similar pathways and networks with comparable effort, regardless of the reward type.

#### Patients vs. community sample

When comparing patients and the community sample, the contrast SZ > CS showed increased couplings in the MID task between the right SMA, associated with planning and preparation of movement (74–76), and the right and left SFG, associated with working memory, self-awareness, and higher cognitive functions (77–79). The increased couplings between these areas might indicate that the MID task is more demanding for patients, requiring a greater involvement of working memory and action planning.

The decreased couplings observed between the parietal operculum, which contains inferior portions of the precentral and postcentral gyri and is involved in primary somatosensory and motor function (80), and the SMA might account for the overall slower reaction times observed in SZ on the behavioral level. This holds true also for decreased couplings between the left pallidum, playing an important role in inhibition and excitation of motor activity (81, 82), and parts of the right and left cerebellum. This could be attributed not only to the disease itself but also to the treatment with antipsychotic drugs, which block striatal D2 receptors in the pallidum, resulting in decreased neural connectivity with corresponding regions.

In summary, when individuals with schizophrenia perform a reward paradigm (MID), apart from slower response times, they exhibit similar behavioral patterns but show differences in neuronal connectivity compared with healthy subjects. The increased couplings might indicate compensatory mechanisms, while the couplings of reduced intensity associated with motor movement could underlie motor deceleration.

Thus, within the patient group, the contrast of MID vs. SID reveals the involvement of regions that have been found to exhibit abnormalities in reward processing in previous studies of individuals with schizophrenia compared with healthy individuals, such as the ACC, the VS, and frontal regions (24, 25, 29, 83). These differences in the two reward paradigms did not manifest in fMRI results (see Supplementary material C) but were evident in the functional connectivity analysis, supporting the notion that schizophrenia is characterized more by disrupted interactions between brain systems rather than isolated focal brain abnormalities.

It is noteworthy that reduced functional connectivity was observed within the patient group during the SID task compared with the MID task, whereas no differences were found in the contrast of patients vs. healthy individuals for the SID paradigm.

The expected discrimination of cue stimuli was less pronounced than expected in the SID paradigm for both samples. While both groups responded equally quickly across all reward levels in the SID task, healthy individuals exhibited overall faster reactions than patients with schizophrenia. Accordingly, no differences in neuronal connectivity were found between healthy individuals and patients in the SID task. Furthermore, no differences in neuronal connectivity between the two paradigms were found in healthy individuals. However, patients exhibited differences in neuronal connectivity depending on the paradigm used, which also resulted in differences between patients and healthy individuals in the MID task, primarily affecting motor areas.

Regarding functional connectivity, our study revealed distinct coupling patterns in both the monetary incentive delay (MID) and social incentive delay (SID) tasks, as well as between patients with schizophrenia and healthy controls. These findings suggest that the neural networks involved in reward processing and connectivity differ depending on the specific task and the presence of schizophrenia.

## Conclusion

The findings of this study highlight the complexity of reward processing in individuals with schizophrenia, suggesting that the functioning of reward systems is not simply disturbed or undisturbed, but rather varies depending on the specific reward paradigm used. Patients with schizophrenia who were in the post-acute phase and treated with second-generation antipsychotics exhibited similar behavioral response patterns to a community sample, with the main difference being slower reaction times. However, despite these behavioral similarities, the underlying neural activations and connectivity patterns differed between the two groups. Therefore, further investigation of the neural mechanisms involved in reward processing is warranted.

The CONN functional connectivity toolbox seems promising for detecting neural networks or connections. To identify these reward-dependent neural networks, the use of EEG-based functional connectivity [e.g., theta-band connectivity (84)], analysis of oscillatory neuronal activity [e.g., theta and high-beta frequencies (85)], and simultaneous EEG-fMRI recordings (86) would also be conceivable for further studies as it allows for better temporal resolution.

Our data suggest that perturbations in reward processing do not imply that underlying connectivities are “destroyed”. Instead, the extent of obvious impairment appears to depend on several factors such as the task or paradigm set, in this case, MID or SID, the type of medication (2, 18), or the severity and phase of the disorder. While patients with severe positive symptoms of schizophrenia tend to overestimate non-predictive cues (2), our patients exhibited a lesser degree of positive symptoms. As a result, we were able to show that patients suffering from schizophrenia are indeed capable of displaying goal-directed, “wanting” inclusive behavior in response to reward-associated stimuli, depending on the aforementioned factors. This indicates that reducing the concept of impaired “wanting” with intact “liking” in individuals with schizophrenia does not fully capture the complexity of possible behavioral and connectivity patterns.

Altered connections in expected brain regions were found within the patient group, depending on the chosen paradigm, but no significant differences were observed when compared to healthy individuals. Disease-related difficulties in responding adequately to reward expectations appear to be more pronounced in the SID paradigm than in the MID paradigm. However, to avoid premature conclusions, the presentation of each paradigm should also be taken into account.

During the SID task, patients responded faster than in the MID task, even on the no reward level, but showed no decrease in reaction times with increasing reward levels, indicating that they were unable to distinguish between the different reward levels, which might be an indication of aberrant salience. It is also conceivable that during the SID paradigm, a high reward incentive was present at each reward level.

Possibly, during monetary reward, the inhibitory potential of the cerebellum for the suppression of aberrant stimuli is better utilized across the different reward levels within the patient sample, indicating that irrelevant stimuli are better suppressed in the MID than in the SID. This raises the question of why this is not the case with social rewards. Perhaps there is no inhibition, but constant activation across different reward levels, as the desire to perceive the different facial expressions outweighs the reward stimulus of strong laughing faces, making discrimination between different reward levels unfeasible. This behavioral observation may align with the results of the connectivity analyses.

### Limitations

There are several limitations to consider. In the present study, both the patient and control groups exhibited no significant decrease in reaction times with increasing expected reward magnitude at the behavioral level. Notably, in contrast to Spreckelmeyer et al. (47), the SID paradigm did not demonstrate a significant influence of reward amount on reaction time.

Another limitation that complicates the interpretation of the results is the small sample size, preventing subgroup analysis. In future investigations, a larger sample size would be beneficial, allowing for gender-based, illness-phase/psychopathology-based, and medication-based subgroup analyses. Additionally, incorporating psychophysiological interactions might yield further insights into the data. To more accurately represent the experimental design, we opted for a three-way repeated-measures ANOVA. Nevertheless, it remains uncertain whether this analysis adequately addresses different types of rewards and their underlying valences.

It is also plausible that task processing within the fMRI scanner influenced reward processing in both groups, contributing to impaired stimulus discrimination. Unfortunately, to the best of our knowledge, no prior study has utilized the SID paradigm with schizophrenia patients, resulting in a lack of comparative data.

## Data availability statement

The raw data supporting the conclusions of this article will be made available by the authors, without undue reservation.

## Ethics statement

The studies involving humans were approved by Ethics Committee of the Medical Faculty of the Justus Liebig University of Giessen. The studies were conducted in accordance with the local legislation and institutional requirements. The participants provided their written informed consent to participate in this study.

## Author contributions

BH, GS, BG, and JI contributed to conception and design of the study. JI organized the database. BH, DL, and JI performed the statistical analysis. BH and JI wrote the first draft of the manuscript. GS and DL wrote sections of the manuscript. All authors contributed to manuscript revision, read, and approved the submitted version.
