# Supplementary material for: Functional connectivity in a monetary and social incentive delay task in medicated patients with schizophrenia
Source: Front Psychiatry. 2023 Aug 30;14:1200860. doi: 10.3389/fpsyt.2023.1200860 (PMC10498543; doi:10.3389/fpsyt.2023.1200860)
Supplement: Supplementary file 1 [file Data_Sheet_1.docx]

| **Supplement A. SZ – MID > SID. Seed analysis, ROI-to-ROI connections.** | | | | | | | |
| --- | --- | --- | --- | --- | --- | --- | --- |
|  |  |  |  |  |  |  |  |
| **Seed** | **ACC** | **Statistics** |  |  | **p-uncorr** | **p-FDR** | **p-FWE** |
|  |  |  |  |  |  |  |  |
|  |  | F(5)(18) | = | 3.32 | 0.0268 | 0.3394 |  |
|  |  | Intensity | = | 74.06 | 0.0002 | 0.0070 | 0.0237 |
|  |  | Size | = | 25 | 0.0006 | 0.0217 | 0.0682 |
|  |  |  |  |  |  |  |  |
| **ACC** | **Brain Stem** | T(22) | = | 4.51 | 0.0002 | 0.0233 |  |
|  | **Cereb10 R** | T(22) | = | 4.02 | 0.0006 | 0.0357 |  |
|  | **Cereb10 L** | T(22) | = | 3.71 | 0.0012 | 0.0366 |  |
|  | **Cereb3 r** | T(22) | = | 3.42 | 0.0025 | 0.0437 |  |
|  | **Cereb6 L** | T(22) | = | 3.40 | 0.0026 | 0.0437 |  |
|  | **Cereb45 R** | T(22) | = | 3.25 | 0.0036 | 0.0492 |  |
|  |  |  |  |  |  |  |  |
|  | **IC L** | T(22) | = | -3.89 | 0.0008 | 0.0357 |  |
|  | **pSTG L** | T(22) | = | -3.67 | 0.0014 | 0.0366 |  |
|  | **pSMG L** | T(22) | = | -3.58 | 0.0017 | 0.0378 |  |
|  | **FP L** | T(22) | = | -3.34 | 0.0030 | 0.0448 |  |
| (L=left; R=right; ACC=anterior cingulate cortex; Cereb=Cerebellum; FP=frontal pole; IC=insular cortex; pSMG= posterior supramarginal gyrus; pSTG=posterior superior temporal gyrus; uncorr=uncorrected) | | | | | | | |
