# Supplementary material for: Functional connectivity in a monetary and social incentive delay task in medicated patients with schizophrenia
Source: Front Psychiatry. 2023 Aug 30;14:1200860. doi: 10.3389/fpsyt.2023.1200860 (PMC10498543; doi:10.3389/fpsyt.2023.1200860)
Supplement: Supplementary file 2 [file Data_Sheet_2.docx]

| **Supplement B. SZ > CS – MID. Seed analysis, ROI-to-ROI connections.** | | | | | | | |
| --- | --- | --- | --- | --- | --- | --- | --- |
|  |  |  |  |  |  |  |  |
| **Seed** | **SMA R** | **Statistics** |  |  | **p-uncorr** | **p-FDR** | **p-FWE** |
|  |  |  |  |  |  |  |  |
|  |  | F(10)(35) | = | 1.98 | 0.0666 | 0.2812 |  |
|  |  | Intensity | = | 71.27 | 0.0009 | 0.0354 | 0.0895 |
|  |  | Size | = | 27 | 0.0011 | 0.0430 | 0.1090 |
|  |  |  |  |  |  |  |  |
| **SMA R** | **SFG L** | T(44) | = | 3.83 | 0.0004 | 0.0299 |  |
|  | **SFG R** | T(44) | = | 3.75 | 0.0005 | 0.0299 |  |
|  |  |  |  |  |  |  |  |
|  | **CO R** | T(44) | = | -3.66 | 0.0007 | 0.0299 |  |
|  |  |  |  |  |  |  |  |
|  |  |  |  |  |  |  |  |
| **Seed** | **aSMG L** | **Statistics** |  |  | **p-uncorr** | **p-FDR** | **p-FWE** |
|  |  |  |  |  |  |  |  |
|  |  | F(10)(35) | = | 2.16 | 0.0458 | 0.2812 |  |
|  |  | Intensity | = | 63.74 | 0.0022 | 0.0422 | 0.1867 |
|  |  | Size | = | 24 | 0.0031 | 0.0539 | 0.2474 |
|  |  |  |  |  |  |  |  |
| **aSMG L** | **IC R** | T(44) | = | -4.13 | 0.0002 | 0.0213 |  |
|  |  |  |  |  |  |  |  |
|  |  |  |  |  |  |  |  |
| **Seed** | **Pallidum L** | **Statistics** |  |  | **p-uncorr** | **p-FDR** | **p-FWE** |
|  |  |  |  |  |  |  |  |
|  |  | F(10)(35) | = | 2.62 | 0.0172 | 0.2178 |  |
|  |  | Intensity | = | 53.23 | 0.0075 | 0.0438 | 0.4501 |
|  |  | Size | = | 20 | 0.0112 | 0.0608 | 0.5738 |
|  |  |  |  |  |  |  |  |
| **Pallidum L** | **Cereb45 R** | T(44) | = | -3.97 | 0.0003 | 0.0214 |  |
|  | **Cereb45 L** | T(44) | = | -3.91 | 0.0003 | 0.0214 |  |
|  |  |  |  |  |  |  |  |
|  |  |  |  |  |  |  |  |
| **Seed** | **SMA L** | **Statistics** |  |  | **p-uncorr** | **p-FDR** | **p-FWE** |
|  |  |  |  |  |  |  |  |
|  |  | F(10)(35) | = | 1.99 | 0.0645 | 0.2812 |  |
|  |  | Intensity | = | 52.62 | 0.0081 | 0.0438 | 0.4692 |
|  |  | Size | = | 20 | 0.0112 | 0.0608 | 0.5738 |
|  |  |  |  |  |  |  |  |
| **SMA L** | **CO R** | T(44) | = | -3.87 | 0.0004 | 0.0480 |  |
| (L=left; R=right; Cereb=Cerebellum; CO=central opercular cortex; IC=insular cortex; SFG=superior frontal gyrus; SMA=supplementary motor areal; aSMG=anterior supramarginal gyrus; uncorr=uncorrected) | | | | | | | |
