# Supplementary material for: Functional connectivity in a monetary and social incentive delay task in medicated patients with schizophrenia
Source: Front Psychiatry. 2023 Aug 30;14:1200860. doi: 10.3389/fpsyt.2023.1200860 (PMC10498543; doi:10.3389/fpsyt.2023.1200860)
Supplement: Supplementary file 3 [file Data_Sheet_3.docx]

Supplement C. fMRI data and analysis.

***Method***

*fMRI preprocessing*

The imaging data were analyzed using SPM8 (Statistical Parametric Mapping, Wellcome Department of Imaging Neuroscience, London, 2009; https://www.fil.ion.ucl.ac.uk/spm/software/spm8/) implemented in MATLAB 7.0 (Mathworks Inc., Sherborn, MA, USA). After discarding the first three volumes to allow for magnetic saturation effects, several preprocessing steps were performed. The images were corrected for differences in slice acquisition time (TA = 2.7067, reference slice = 15) and subject movement (twice the voxel size at maximum). Fieldmap correction was applied to reduce distortions caused by field inhomogenities. To compensate for variation in brain structures among different participants, the functional images were coregistered to the participant’s anatomical image (using normalized mutual information function) and converted to standard space by fitting to the Montreal Neurological Institute (MNI; (Lancaster et al., 2007)) reference brain. Voxel size of the rewritten images was 3mm x 3mm x 3mm. Afterwards, the resulting images were smoothed using 9 mm FWHM Gaussian Kernel.

*fMRI analyses*

A random-effects, event-related statistical analysis (Josephs et al., 1997) was performed with SPM8 in a two-level procedure. At the first level, a separate general linear model (GLM) was specified for each participant. Task-related changes in BOLD signal were estimated at each voxel by modelling the onsets of each trial as delta functions convolved with a hemodynamic response function (HRF). Both tasks (MID, SID) with their four levels of hypothesized reward magnitude were modelled as separate regressors. Regression coefficients for all regressors were estimated using ordinal least squares within SPM8 (Friston et al., 1995). Condition-specific effects were tested using the appropriate linear contrasts of the parameter estimates for the HRF regressors of all conditions. The resulting set of voxel values for each contrast constitutes a statistical parametric map. For both reward types, a “trend” contrast displaying the four increasing reward levels (-3, -1, 1, 3) was comprised as well as a contrast opposing the three reward levels between the reward types (SID_reward_ > MID_reward_). To examine group effects each person’s contrast values were analysed on “second level”. In a first step the data was analyzed for both groups separately, then the data of both groups were compared (SZ > CS, CS > SZ).

Therefore, cluster analyses (*p*<.001 uncorrected, cluster size ≥ 100 voxels), reporting the FWE corrected p-value, MNI coordinates for the peak voxels and corresponding brain regions.

, as well as, region-of-interest-analyses were performed on voxel level (*t*-tests for one sample and *t*-tests for two samples; *p* < 0,05, FWE corrected). The used regions of interest (ROI) are associated with reward processing and reward anticipation (Delvecchio et al., 2013; Esslinger et al., 2013; Gruber et al., 2014; Minzenberg et al., 2009; Spreckelmeyer et al., 2009) and included the VS left and right, the insular cortex (IC, left and right), the cingulate cortex (anterior, ACC, and posterior, PCC), the thalamus (left and right), and the supplementary motor areal (SMA, left and right). If available, ROI-masks were derived from the Wake Forest University (WFU) PickAtlas-Toolbox Version 2.4. Peak-voxel (*p* < .05, FWE corrected), MNI coordinates and corresponding brain regions were reported.

For the VS, a voxel mask of the publication-based, probabilistic MNI atlas was used (Fox & Lancaster, 2002; Nielsen & Hansen, 2002); available at http://hendrix.imm.dtu.dk/services/jerne/ninf/voi/index-alphabetic.html). Based on the given specification and coordinates a binary voxel mask was created for the left and right hemisphere with a probability threshold of p = 0.75 (see (Koch et al., 2015; Schlagenhauf et al., 2008)).

Results

***fMRI***

At cluster level we found wide networks of brain areas sensitive to the increasing level of monetary as well as social rewards (see Table A).

| **Table A. Brain regions showing proportional activation to increasing anticipated reward for SZ and CS.** | | | | | | | |
| --- | --- | --- | --- | --- | --- | --- | --- |
|  | |  |  | | |  |  |
| **Brain regions** | |  | **MNI-Coordinates** | | |  |  |
|  | |  | x | y | z | Peak z-score | Cluster size |
| **MID CS pos** | |  |  |  |  |  |  |
| L ACC |  | | -3 | 11 | 22 | 3.39 | 65*** |
| R SMA |  | | 9 | 2 | 61 | 3.63 | 147*** |
| L MCC |  | | -6 | -22 | 25 | 3.13 | 68*** |
| R MCC |  | | 12 | -1 | 31 | 3.06 | 12*** |
| R MOG |  | | 30 | -97 | 1 | 2.85 | 39** |
| L Insular cortex |  | | -39 | 17 | -5 | 2.64 | 19** |
| **MID CS neg** | |  |  |  |  |  |  |
| R Precuneus | |  | 9 | -52 | 37 | 4.49 | 518*** |
| R Angular gyrus | |  | 48 | -58 | 34 | 4.35 | 223*** |
| R Postcentral gyrus | |  | 27 | -25 | 34 | 3.42 | 36*** |
| R Medial frontal gyrus | |  | 9 | 56 | 10 | 2.89 | 31** |
| L Medial frontal gyrus | |  | -9 | 47 | 4 | 2.82 | 35** |
| L Angular gyrus | |  | -51 | -64 | 31 | 2.71 | 29** |
| L Middle frontal gyrus | |  | -24 | 17 | 31 | 2.65 | 11** |
| **SID CS pos** | |  |  |  |  |  |  |
| --- | |  |  |  |  |  |  |
| **SID CS neg** | |  |  |  |  |  |  |
| R SFG | |  | 18 | 41 | 25 | 3.78 | 220*** |
| L MTG | |  | -63 | -28 | 1 | 3.44 | 28*** |
| L Middle frontal gyrus | |  | -21 | 29 | 25 | 3.30 | 95*** |
| R STG | |  | 54 | -34 | 4 | 3.17 | 55*** |
|  | |  |  |  |  |  |  |
| **MID SZ pos** | |  |  |  |  |  |  |
| R SMA | |  | 12 | 5 | 64 | 3.62 | 60 |
| L SMA | |  | -15 | -4 | 61 | 2.97 | 20 |
| **MID SZ neg** | |  |  |  |  |  |  |
| L Thalamus | |  | -3 | -25 | 10 | 3.11 | 53 |
| L Precuneus | |  | -3 | -61 | 49 | 3.05 | 232 |
| L ITG | |  | -45 | -61 | -8 | 2.93 | 25 |
| L MTG | |  | -51 | -28 | -11 | 2.88 | 11 |
| R MTG | |  | 63 | -10 | -17 | 2.87 | 14 |
| R Cuneus | |  | 21 | -67 | 25 | 2.83 | 15 |
| L Cuneus | |  | -3 | -70 | 25 | 2.73 | 27 |
| R Parahippocampal gyrus | |  | 24 | -16 | -20 | 2.67 | 10 |
| **SID SZ pos** | |  |  |  |  |  |  |
| R Angular gyrus | |  | 24 | -49 | 34 | 3.48 | 200 |
| R ITG | |  | 42 | -4 | -29 | 3.42 | 13 |
| R Nucleus caudate | |  | 9 | 20 | 7 | 2.97 | 13 |
| L Inferior parietal lobule | |  | -24 | -25 | 34 | 2.91 | 51 |
| L Nucleus Caudate | |  | -9 | 17 | 10 | 2.86 | 32 |
| **SID SZ neg** | |  |  |  |  |  |  |
| R SMG | |  | 63 | -49 | 28 | 3.29 | 34 |
| R ITG | |  | 51 | -70 | -8 | 3.18 | 43 |
| L MTG | |  | -51 | -73 | 13 | 3.04 | 109 |
| L SMA | |  | 0 | 20 | 55 | 3.03 | 101 |
| L IFG, orbital part | |  | -42 | 23 | -14 | 2.93 | 22 |
| R Precentral gyrus | |  | 57 | -13 | 49 | 2.90 | 19 |
| R SMG | |  | 66 | -16 | 25 | 2.74 | 12 |
| L SFG | |  | -15 | 56 | 25 | 2.69 | 22 |
| L Postcentral gyrus | |  | -60 | -10 | 37 | 2.69 | 19 |
| L SFG, medial orbital | |  | -3 | 56 | -11 | 2.69 | 10 |
| L Precuneus | |  | -3 | -55 | 40 | 2.59 | 13 |
| ***p<0.001 uncorrected, **p<0.01 uncorrected (L=left; R=right; ACC=anterior cingulate cortex; IFG=inferior frontal gyrus; ITG=inferior temporal gyrus; MCC=midcingulate cortex; MFG=medial frontal gyrus; MOG=middle occipital gyrus; MTG=middle temporal gyrus; SFG=superior frontal gyrus; SMA=supplementary motor areal; SMG=supramarginal gyrus; STG=superior temporal gyrus) | | | | | | | |

ROI-Analyses were performed for the following regions: VS (left and right), IC (left and right), ACC and PCC, thalamus (left and right), SMA (left and right). We considered MID_trend_, SID_trend_ and MID_reward_ > SID_reward_ separately for both groups as well as for the group comparisons.

The above mentioned ROI-Analyses showed no significant activation differences, neither within the analyzed groups, nor between patients and controls.

fMRI

At cluster level we found wide networks of brain areas sensitive to the increasing level of monetary as well as social rewards (see also Table 3) (broadly) similar to the network of brain areas detected by Spreckelmeyer et al. (Spreckelmeyer et al., 2009). These findings confirmed, along with the behavioural results, that the used paradigms stimulate reward processing networks comparable to previous studies.

Previous studies have demonstrated an association between disturbed reward-related learning and disturbed VS activation (Jensen et al., 2008; Schlagenhauf et al., 2014). Abnormalities in midbrain, insula and amygdala (Corlett et al., 2007; Gradin et al., 2013; Juckel et al., 2006; Romaniuk et al., 2010; Waltz et al., 2009) suggest that alterations in the mesolimbic dopamine system underlie deficits in reward based learning. In opposite to these findings, we did not find neural activation differences, neither for the respective paradigms nor for the conducted groups.

Literature

Corlett, P. R., Murray, G. K., Honey, G. D., Aitken, M. R. F., Shanks, D. R., Robbins, T. W., Bullmore, E. T., Dickinson, A., & Fletcher, P. C. (2007). Disrupted prediction-error signal in psychosis: Evidence for an associative account of delusions. *Brain*, *130*(9), 2387–2400. https://doi.org/10.1093/brain/awm173

Delvecchio, G., Sugranyes, G., & Frangou, S. (2013). Evidence of diagnostic specificity in the neural correlates of facial affect processing in bipolar disorder and schizophrenia: a meta-analysis of functional imaging studies. *Psychological Medicine*, *43*(3), 553–569. https://doi.org/10.1017/S0033291712001432

Esslinger, C., Braun, U., Schirmbeck, F., Santos, A., Meyer-Lindenberg, A., Zink, M., & Kirsch, P. (2013). Activation of midbrain and ventral striatal regions implicates salience processing during a modified beads task. *PLoS ONE*, *8*(3), e58536. https://doi.org/10.1371/journal.pone.0058536

Fox, P. T., & Lancaster, J. L. (2002). Mapping context and content: The BrainMap model. *Nature Reviews Neuroscience*, *3*(4), 319–321. https://doi.org/10.1038/nrn789

Friston, K. J., Ashburner, J., Frith, C. D., Poline, J.-B., Heather, J. D., & Frackowiak, R. S. J. (1995). Spatial Registration and Normalization of Images. *Human Brain Mapping*, *2*, 165–189.

Gradin, V. B., Waiter, G., O’Connor, A., Romaniuk, L., Stickle, C., Matthews, K., Hall, J., & Steele, J. D. (2013). Salience network-midbrain dysconnectivity and blunted reward signals in schizophrenia. *Psychiatry Research - Neuroimaging*, *211*(2), 104–111. https://doi.org/10.1016/j.pscychresns.2012.06.003

Gruber, O., Santuccione, A. C., & Aach, H. (2014). Magnetic resonance imaging in studying schizophrenia, negative symptoms, and the glutamate system. *Frontiers in Psychiatry*, *5*(April), 1–11. https://doi.org/10.3389/fpsyt.2014.00032

Jensen, J., Willeit, M., Zipursky, R. B., Savina, I., Smith, A. J., Menon, M., Crawley, A. P., & Kapur, S. (2008). The formation of abnormal associations in schizophrenia: neural and behavioral evidence. *Neuropsychopharmacology*, *33*, 473–479. https://doi.org/10.1038/sj.npp.1301437

Josephs, O., Turner, R., & Friston, K. (1997). Event-Related fMRI. *Hum. Brain Mapping*, *5*, 243–248.

Juckel, G., Schlagenhauf, F., Koslowski, M., Wüstenberg, T., Villringer, A., Knutson, B., Wrase, J., & Heinz, A. (2006). Dysfunction of ventral striatal reward prediction in schizophrenia. *NeuroImage*, *29*, 409–416. https://doi.org/10.1016/j.neuroimage.2005.07.051

Koch, S. P., Hägele, C., Haynes, J. D., Heinz, A., Schlagenhauf, F., & Sterzer, P. (2015). Diagnostic classification of schizophrenia patients on the basis of regional reward-related fMRI signal patterns. *PLoS ONE*, *10*(3). https://doi.org/10.1371/journal.pone.0119089

Minzenberg, M. J., Laird, A. R., Thelen, S., Carter, C. S., & Glahn, D. C. (2009). Meta-analysis of 41 functional neuroimaging studies of executive function in schizophrenia. *Archives of General Psychiatry*, *66*(8), 811–822. https://doi.org/10.1001/archgenpsychiatry.2009.91.Meta-analysis

Nielsen, F. Å., & Hansen, L. K. (2002). Modeling of activation data in the BrainMap^TM^ database: Detection of outliers. *Human Brain Mapping*, *15*(3), 146–156. https://doi.org/10.1002/hbm.10012

Romaniuk, L., Honey, G. D., L King, J. R., Whalley, H. C., McIntosh, A. M., Levita, L., Hughes, M., Johnstone, E. C., Day, M., Lawrie, S. M., & Hall, J. (2010). Midbrain Activation During Pavlovian Conditioning and Delusional Symptoms in Schizophrenia. *Arch Gen Psychiatry*, *67*(12), 1246–1254. www.biopac.com

Schlagenhauf, F., Huys, Q. J. M., Deserno, L., Rapp, M. A., Beck, A., Heinze, H. J., Dolan, R., & Heinz, A. (2014). Striatal dysfunction during reversal learning in unmedicated schizophrenia patients. *NeuroImage*, *89*, 171–180. https://doi.org/10.1016/j.neuroimage.2013.11.034

Schlagenhauf, F., Juckel, G., Koslowski, M., Kahnt, T., Knutson, B., Dembler, T., Kienast, T., Gallinat, J., Wrase, J., & Heinz, A. (2008). Reward system activation in schizophrenic patients switched from typical neuroleptics to olanzapine. *Psychopharmacology*, *196*, 673–684. https://doi.org/10.1007/s00213-007-1016-4

Spreckelmeyer, K. N., Krach, S., Kohls, G., Rademacher, L., Irmak, A., Konrad, K., Kircher, T., & Gründer, G. (2009). Anticipation of monetary and social reward differently activates mesolimbic brain structures in men and women. *Social Cognitive and Affective Neuroscience*, *4*(2), 158–165. https://doi.org/10.1093/scan/nsn051

Waltz, J. A., Schweitzer, J. B., Gold, J. M., Kurup, P. K., Ross, T. J., Jo Salmeron, B., Rose, E. J., McClure, S. M., & Stein, E. A. (2009). Patients with schizophrenia have a reduced neural response to both unpredictable and predictable primary reinforcers. *Neuropsychopharmacology*, *34*(6), 1567–1577. https://doi.org/10.1038/npp.2008.214
